# Supplementary figures and images for: Exogenous recombinant N-acetylgalactosamine-4-sulfatase (Arylsulfatase B; ARSB) inhibits progression of B16F10 cutaneous melanomas and modulates cell signaling
Source: Biochim Biophys Acta Mol Basis Dis. Author manuscript; Available in PMC 2024 Jul 23. (PMC11265800; doi:10.1016/j.bbadis.2023.166913)

## Slide 1
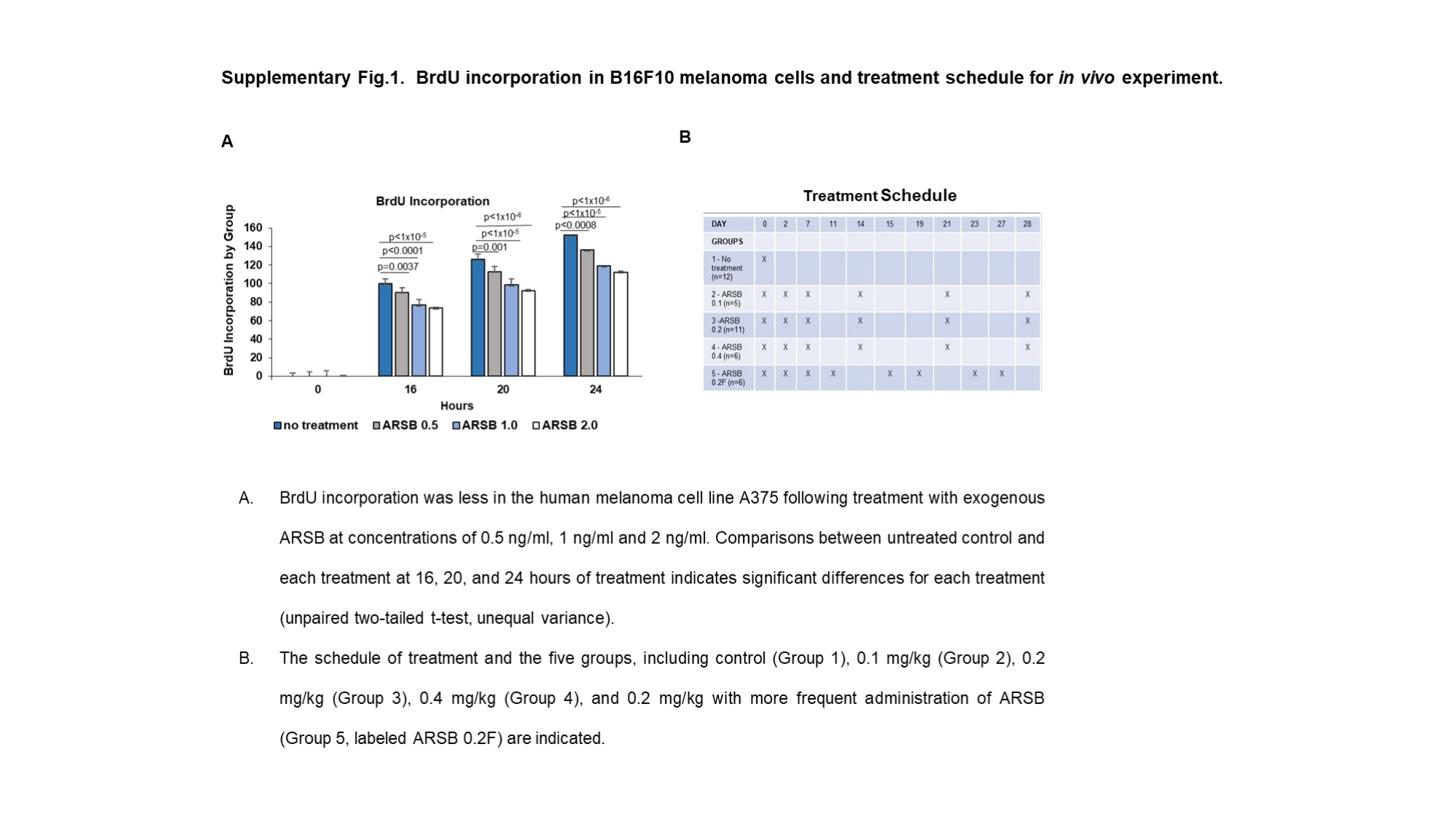

Supplement: Supplementary Fig.1 [file NIHMS1994486-supplement-Supplementary_Fig_1.pptx]
